# Language-based inference generation under working memory load: the role of schizotypal traits in jumping to conclusions

**DOI:** 10.3389/fpsyt.2025.1660324

**Published:** 2026-01-22

**Authors:** Emily C. Gann, Gabby Sandlin, Yanyu Xiong, Chuong Bui, Sharlene D. Newman

**Affiliations:** 1 Department of Psychology, The University of Tulsa, Tulsa, OK, United States; 2 Alabama Life Research Institute, The University of Alabama, Tuscaloosa, AL, United States

**Keywords:** schizophrenia, theory of mind (ToM), speech production, circular inference, disorganized schizotypal traits

## Abstract

**Background and hypothesis:**

Individuals with schizophrenia often exhibit language abnormalities and impairments in Theory of Mind (ToM). These difficulties may stem from underlying cognitive processes such as a tendency to jump to conclusions (JTC), making decisions without sufficient external evidence, and disruptions in circular inference, which can produce atypical beliefs, impaired probabilistic decision-making, and heightened perceptions of visual ambiguity. However, it remains unclear whether similar impairments occur in healthy individuals who display non-clinical schizotypal personality traits.

**Study design:**

The present study examined JTC through inference generation and its association with schizotypal traits. A total of 532 participants completed the Schizotypal Personality Questionnaire–Brief Revised (SPQ-BR) and were audio-recorded while narrating a nine-frame comic strip. A between-subjects working memory (WM) manipulation was used to assess the effect of cognitive load. Inference generation was evaluated by independent raters who manually annotated the speech transcripts.

**Study results:**

WM load reliably increased the number of inferred events produced, whereas schizotypal traits alone were not significantly associated with inferred events. Instead, WM load moderated this relationship: disorganized traits predicted more inferred events only under WM load. For visual events, disorganized traits demonstrated a quadratic association, and WM load again moderated this pattern, with quadratic effects emerging only when WM load was absent. Overall, WM load played a central role in shaping how disorganized schizotypal traits related to both inferred and visual event production.

**Conclusions:**

These findings indicate that increased JTC tendencies can emerge even in the absence of clinically significant schizophrenia symptoms and that disorganized traits may contribute to disruptions in circular inference mechanisms. The results also underscore the utility of analyzing speech production as a method for investigating inference generation in future research.

## 1 Introduction

Schizotypal traits are a set of commonly found personality traits that resemble schizophrenia but exist at a subclinical threshold (1). Like schizophrenia, schizotypal traits are characterized by positive, negative, and disorganized domains (2). Positive traits involve characteristics ranging from magical beliefs to delusions. Negative traits include deficits such as anhedonia (lack of interest and pleasure in life’s activities), blunted affect, and low energy or motivation. Finally, disorganized schizotypy encompasses disruptions in speech, behavior, and emotions (2).

Some research suggests schizotypal traits may be a cognitive and biological predisposition to development of schizophrenia (3, 4). The presence of schizotypal traits has been associated with cognitive dysfunction (e.g., attention and cognitive inhibition (5–7) and language abnormalities (8–10). Researchers have investigated the mechanisms underlying unusual language production, finding that schizotypy may be linked to differences in how meaningful stimuli activate related concepts in semantic memory (11). Some researchers hypothesize that the unusual associations observed in schizotypy reflect simultaneously activated concepts in the brain, supporting a model of semantic long-term memory (12–15).

The process of jumping to conclusions (JTC) has been observed in schizophrenia patients and may be associated with underlying impairments of Theory of Mind (ToM) that have been well-established within schizophrenia (16–19). It is theorized that individuals with schizophrenia may make decisions based on limited evidence when required to collect information before making an inference (20). Studies on JTC have found that schizophrenia patients produce more inferences than healthy controls and may be overconfident in an event’s likelihood when confronted with potentially disconfirmatory information (21, 22). These findings suggest the involvement of a reasoning abnormality, potentially coexisting with perceptual abnormalities (23). Additionally, knowledge corruption is significantly greater in schizophrenia, leading to an increased likelihood of false-positive and false-negative judgments (24).

JTC may be associated with circular inferencing. The brain functions as a hierarchical system that makes probabilistic inferences (25–28). Circular inference occurs when sensory data are corrupted due to prior information or vice versa, leading individuals to see what they expect and expect what they see or a mix of both (29). The circular inference framework and its neural circuits may be impaired, leading to atypical beliefs that impact decision-making and heightened visual perception of ambiguity (28). Further, research suggests that probabilistic inference of future events is linked to an individual’s ability to maintain recent past experiences in working memory (30).

Performance tasks such as the beads task (21) are commonly used to assess jumping to conclusions (JTC), yet relatively little research has examined how JTC influences speech production. Therefore, the goal of the current study was to investigate inference-making during story production and to determine whether schizotypal traits are associated with performance. In this task, participants generated a story from a cartoon, with a between-subjects working memory (WM) manipulation: in one condition, the cartoon remained on the screen during production, whereas in the other, it was removed prior to production. To complete the task, participants must construct a situation model—a memory-based representation that includes their interpretation of the cartoon and the inferences drawn from it (31, 32). This demand is especially pronounced in the WM condition, where visual support is absent. Because the situation model necessarily involves inferential processing, we predicted that participants in the WM condition would produce more inferences overall and show a stronger association between inference generation and schizotypy. Additionally, based on prior evidence that individuals with schizophrenia exhibit heightened JTC tendencies, we expected that individuals with higher schizotypal traits would generate more inferences than those with lower trait levels.

## 2 Methods

### 2.1 Participants

532 undergraduate students enrolled at The University of Alabama participated in the study for course credit. The study was approved by The University of Alabama’s Institutional Review Board.

### 2.2 Procedure

Participants were recruited through an advertisement posted on the psychology subject pool website (SONA) and were directed to a Qualtrics survey, where all data were collected. The survey consisted of two parts. In the first part, participants completed demographic questions as well as measures assessing substance use history (alcohol and cannabis) and schizotypal traits. The second part included a picture description task (33) and a story generation task. Audio responses for both tasks were recorded using Phonic, an embedded audio-capture tool integrated within Qualtrics (https://www.phonic.ai/).

### 2.3 Schizotypal traits

Schizotypal traits were assessed using the Schizotypal Personality Questionnaire–Brief Revised (SPQ-BR; 42), a 32-item measure that captures positive, negative, and disorganized dimensions of schizotypy. Although the SPQ-BR was not originally validated using college student samples, O’Hare and Linscott (34) reported that use in convenience samples—such as undergraduate participants—does not compromise the generalizability of the relationships among the first-order SPQ-BR dimensions or their associations with other variables.

### 2.4 Discourse production task

Participants were presented with a nine-frame comic strip consisting of three unnamed characters with by cartoonist Andy B. Childress (http://bubbaworldcomix.com/). The comic requires inference making about the emotions and/or intentions of characters. For the non-WM load group, the comic strip remained on the screen with instructions to press the record button and begin describing the comic. The WM load group was presented the comic on the screen for 1 minute with instructions to study the comic, then was automatically removed and participants then were presented with instructions to press the record button and describe the comic. All participants were instructed that their recording should be between 60 to 90 seconds. Transcripts were automatically transcribed then manually checked for errors.

### 2.5 Discourse analysis

Audio recordings were transcribed using Otter transcription services, and a random subset of transcripts was reviewed for accuracy. Research team members were trained to manually annotate the transcripts using a predefined rubric and were blind to all study conditions and participant characteristics. Annotators identified two types of content (1): references to events or actions explicitly depicted in the cartoon (e.g., “the man is reading a newspaper,” “the man falls to the ground”) and (2) inferred content describing characters’ thoughts, feelings, intentions, or unobserved actions (e.g., “he’s scared of the clown,” “the two men are planning a prank”; see Appendix for instructions and examples). In addition, annotators coded all mentalizing verbs, including cognitive, volitional, and emotional verbs.

Each transcript was annotated by two independent raters. A third reviewer conducted an inter-rater comparison and flagged discrepancies, which were resolved during weekly lab meetings until consensus was reached. Interrater reliability was 0.84. Raters remained fully blind to participant demographics and SPQ-BR scores throughout the discourse analysis.

### 2.6 Statistical analysis

The dependent variables were *inferred events* and *visual events*. The independent variables of interests were schizotypal traits, including positive, negative and disorganized traits. Included as covariates were alcohol use (days/week), cannabis use (days/week), sex (male/female), working status (yes/no), working memory (WM) load (yes/no), and grade point average (GPA). Prior to the primary analyses, we utilized (semiparametric) generalized additive models to examine possible non-linear relationships between schizotypal and inferred events and visual events. Smoothing splines (i.e. nonparametric) were specified for schizotypal traits, alcohol use, cannabis use and GPA. Parametric effects were specified for (categorical variables) sex, employment, and WM load. Poisson was the assumed underlying distribution. The logarithm of the number of words was used as the offset variable. Smoothing parameters were selected by generalized cross validation. Primary analyses were carried out using Poisson regression. To account for overdispersion, robust standard errors were used to for inference. Statistical analyses were performed in SAS/STAT 15.1 software (PROC GAM for generalized additive models, PROC GENMOD for generalized linear models).

## 3 Results

### 3.1 Sample description

The sample included 532 college. One participant who gave an invalid response to the question about GPA (GPA = 80) was removed, reducing the sample to 531. Participants averaged 18.75 years old (± 1.01) (**Table 1**). The youngest was 18 years old, oldest 26. The sample was predominantly female (81.0%) and white (81.5%; African American 121%; other 6.4%). GPA ranged between 0 and 4.33, with an average of 3.36 ( ± .81). About 66.6% were not working for pay, while 33.4% were part-time or full-time employed (the majority was part-time). About 80.2% reported not using cannabis in the past 90 days, 11.3% day a week, and 19.5% two or more days a week. About 40.1% reported not using alcohol in the past 90 days, 25.6% one day a week, and 34.3% two or more days a week. There were 270 participants in the no WM load condition, and 260 in WM load condition. Schizotypal traits were moderately correlated with one another (positive-negative, r =.59; positive-disorganized, r =.52; negative-disorganized, r =.45). Positive traits averaged 19.53 (± 9.81); negative traits 17.43 (± 7.09); and disorganized traits 15.17 (± 5.93).

**Table 1:** Associations between schizotypal traits and inferred and visual events.

	Number of inferred events			Number of visual events		
	b	se	p	b	se	p
Positive	-.012	.011	.268	.000	.002	.957
Positive_sq	.000	.000	.243	_	_	_
Negative	-.018	.013	.188	.003	.002	.151
Negative_sq	.000	.000	.228	_	_	_
Disorganized	.008	.005	.105	.017	.009	.061
Disorganized_sq	_	_	_	-.001	.000	.020
Sex (male)	.037	.063	.556	-.009	.038	.805
GPA	-.012	.032	.698	.002	.016	.895
Working status (yes)	.008	.051	.883	-.062	.026	.017
Alcohol use	.002	.020	.942	-.015	.010	.141
Cannabis use	-.053	.022	.013	.019	.009	.025
Working memory (WM) load	.233	.051	<.001	-.054	.025	.031

*Positive_sq*, *Negative_sq*, and *Disorganized_sq* are the quadratic terms for positive, negative and disorganized traits, respectively.

In the current sample, schizotypal traits (SPQBR) had acceptable internal reliability (positive, McDonald’s ω =.88; negative ω =.85; disorganized traits ω =.82). Based on the Heterotrait-monotrait ratios (HTMT), the traits showed acceptable discriminant validity (positive-negative, HTMT = .63; positive-disorganized.58; negative-disorganized (.51). Schizotypal traits (SPQBR) overall were moderately correlated with 2 other scales of schizotypal signs, that is, the Formal Thoughts Disorder (FTD) and the Perceptual Aberration Scale (PAS) (positive-FTD, r=.63; positive-PAS, r =.45; negative-FTD, r =.58; negative-PAS, r =.31; disorganized-FTD, r =.62; disorganized-PAS, r =.37);.

### 3.2 Associations between schizotypal traits and inferred events

Prior to primary analyses, we utilized a generalized additive model to explore nonlinear relationships between schizotypal traits and inferred events. Results suggested a possible non-linear component for positive traits (p=.037) and negative traits (p=.064) that resembled a quadratic pattern (Appendix, Figure A). In light of this, we fit a Poisson regression with quadratic terms for positive and negative traits. Parameter estimates were presented in **Table 1**. The quadratic relationship between positive traits and inferred events were non-significant (linear term, p=.268; quadratic term, p=.243). The same was true for positive traits (linear term, p=.188; quadratic term, p=.228). The (linear) association between disorganized traits and inferred events was also non-significant (p=.105). Working memory (WM) load was significantly associated with higher inferred events (p<.001).

**Figure 1:**
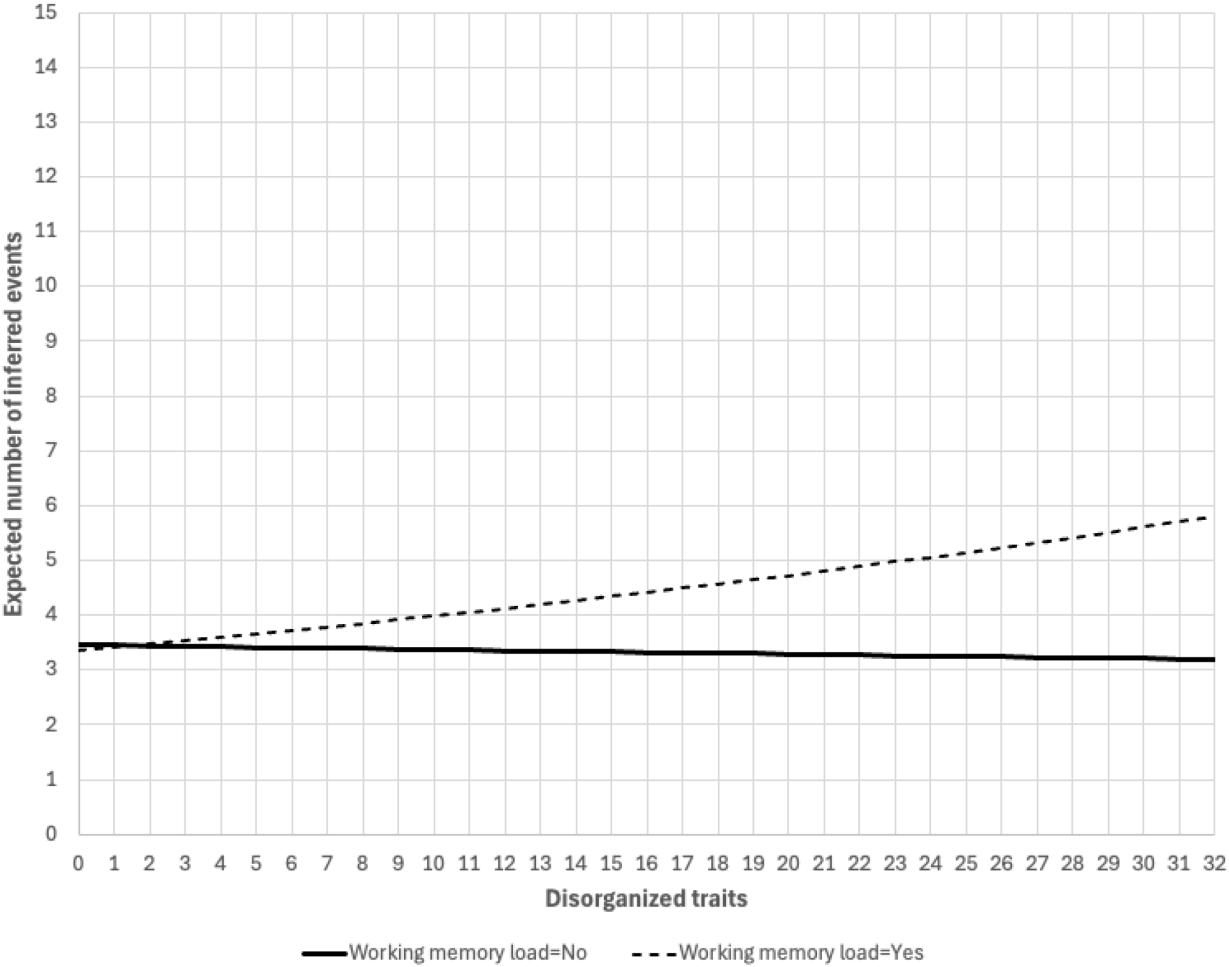
WM load-moderated relationship between disorganized traits and inferred events. Note: Figure was generated using estimates from **Table 2**. Observed values of disorganized traits ranged between 0-32. Positive traits, negative traits, alcohol use, cannabis use, and GPA were fixed at sample averages. Sex was fixed at female and working status at not working.

Building upon the above analyses, we examined if WM load moderated the associations between schizotypal traits and inferred events. The regression model now included the interactions between WM and positive traits (linear term and quadratic term), negative traits (linear and quadratic), disorganized traits (linear). WM loads did not appear to moderate the associations between positive or negative traits and inferred events. On the other hand, WM appeared to moderate the associations between disorganized traits and inferred events (p=.062) (**Table 2**). For the group that did not have WM load, disorganized traits did not appear associated with inferred events (b= -.003, p=.758). For the group that had WM load, disorganized traits was associated with higher inferred events (b=.017, p=.010). **Figure 1** illustrated the WM load-moderated relationships between disorganized traits and inferred events.

**Table 2:** Working memory load-moderated associations between schizotypal traits and inferred and visual events.

	Number of inferred events			Number of visual events		
	b	se	p	b	se	p
WM load	.045	.235	.848	.148	.115	.196
Positive	-.014	.017	.415	.001	.002	.819
Positive * WM	.004	.021	.836	-.001	.003	.734
Positive_sq	.000	.000	.250	_	_	_
Positive_sq * WM	.000	.001	.514	_	_	_
Negative	-.017	.018	.327	.001	.003	.712
Negative * WM	.001	.027	.984	.004	.005	.375
Negative_sq	.001	.001	.283	_	_	_
Negative_sq * WM	.000	.001	.836	_	_	_
Disorganized	-.003	.008	.758	.032	.013	.010
Disorganized * WM	.020	.011	.062	-.036	.017	.031
Disorganized_sq	_	_	_	-.001	.000	.002
Disorganized_sq * WM	_	_	_	.001	.001	.024
Sex (male)	.036	.064	.573	-.010	.039	.802
GPA	-.008	.032	.800	.004	.016	.796
Working status (yes)	-.002	.052	.971	-.063	.026	.015
Alcohol use	.001	.020	.960	-.014	.010	.165
Cannabis use	-.046	.022	.033	.020	.009	.021

*Positive_sq*, *Negative_sq*, and *Disorganized_sq* are the quadratic terms for positive, negative and disorganized traits, respectively.

*, interaction.

### 3.3 Associations between schizotypal traits and visual events

In a similar fashion, we started with a generalized additive model to explore nonlinear relationships between schizotypal traits and visual events. Results suggested a possible non-linear component for disorganized traits (p=.017) that resembled a quadratic pattern (Appendix, Figure B). In light of this, we estimated a Poisson regression with a quadratic term for disorganized traits (**Table 1**). The quadratic relationship between disorganized traits and visual events were significant (linear term, b= .017, p=.061; quadratic term, b= -.001, p=.020). The (linear) relationships between positive and negative traits and visual events were non-significant (p=.957 and p=.151, respectively). Working memory load was significantly associated with lower visual events (p=.031).

**Figure 2:**
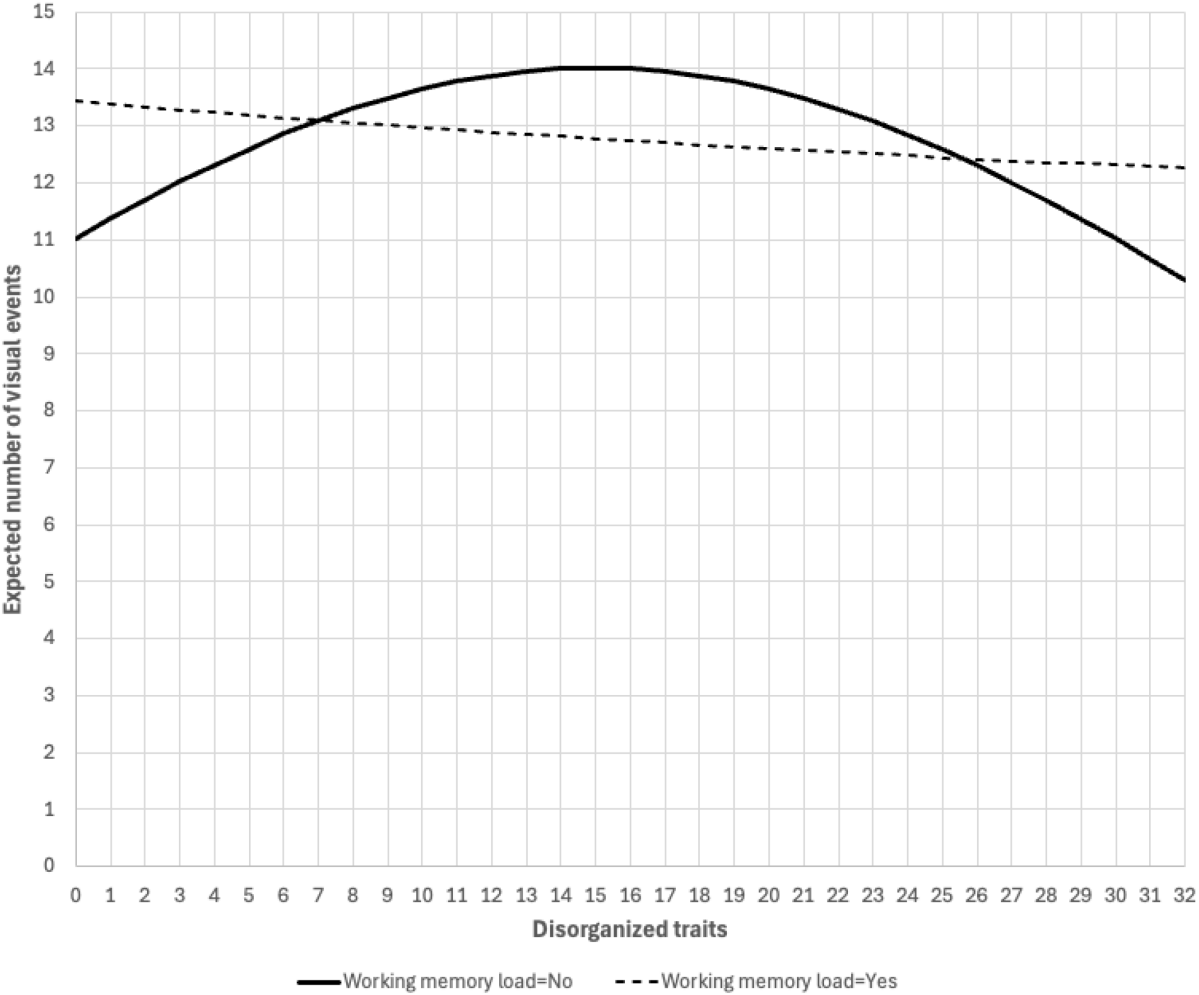
WM load-moderated relationship between disorganized traits and visual events. Note: Figure was generated using estimates from **Table 2**. Observed values of disorganized traits ranged between 0-32. Positive traits, negative traits alcohol use, cannabis use, and GPA were fixed at sample averages. Sex was fixed at female and working status at not working.

We next examined the moderation effects of WM load by incorporating interactions between WM and positive traits (linear term), negative traits (linear), disorganized traits (linear and quadratic). WM loads appeared to moderate both the linear and quadratic terms of disorganized traits (p=.031 and p=.024, respectively). For the group without WM load, the relationship between disorganized traits and visual events appeared to have a quadratic pattern (linear term, b=.032, p=.010; quadratic term, b= -.001 p=.002). On the contrary, for the group with WM load, both the linear and quadratic terms of disorganized traits were non-significant (linear term, b= -.004, p=.743; quadratic term, b= .000 p=.936). **Figure 2** illustrated the WM load-moderated relationships between disorganized traits and visual events. Without WM load, on its lower end, increasing disorganized traits is associated with increasing (expected) visual events. On its higher end, increasing disorganized traits is associated with decreasing (expected) visual events.

## 4 Discussion

The results of the current study demonstrated differences in inference making in individuals with high schizotypal traits. Creating a WM load and pushing participants to create a situation model of the cartoon resulted in reductions in the total number of events described primarily due to describing more visual events when the cartoon is presented on the screen. However, those with the WM load produced a higher proportion of inferred events, as predicted, due to creating a situation model. Additionally, the proportion of inferred events described varied as a function of disorganized traits such that individuals with higher disorganized traits generated more inferred events, but only in the WM load condition.

The situation model includes the interpretation of the scene depicted in text, video or in the case of the current study, the cartoon. It incorporates the concretely depicted visual/spatial information as well as inferred information including the characters’ goals and motivations (35, 36). Research suggests that deficits related to discourse coherence were likely observed in the construction of the situation model (37). The results of the current study support the creation of a situation model when a memory representation of the cartoon is required to be generated. Although the no WM load group produced more total event descriptions, participants in the WM load group produced a higher proportion of inferences. This result also corroborates those reported by Costabile (2016) that demonstrated that narrative construction, like that performed in the current study, elicits the construction of a situation model that incorporates inferences (38).

While speculative, we propose that the observed association between disorganized traits and inferred events in the WM load condition may be consistent with patterns linked to jumping to conclusions (JTC). Disorganized traits are typically associated with difficulties in organizing and regulating thought (39, 40), and they reflect characteristics such as eccentric behavior and atypical speech patterns. Prior work by Krężołek and colleagues (2019) reported that JTC tendencies were related more strongly to disorganization severity than to delusion severity among individuals with schizophrenia (41). Similarly, Jardri et al. (2017) noted that perceptual–cognitive phenomena related to circular inferencing, such as “seeing what we expect” and “expecting what we see,” were associated with disorganization (29).

Although our findings cannot directly confirm these mechanisms, the pattern we observed, where disorganized traits, but not positive or negative traits, showed an association with inference generation, appears broadly consistent with these previous reports. At the same time, alternative explanations remain possible, and additional work is needed to determine whether the processes underlying JTC in clinical samples extend to non-clinical populations. Nevertheless, these results suggest that studying disorganization-related tendencies in non-clinical groups may offer a useful avenue for exploring the cognitive components linked to inference-making.

### 4.1 Limitations

Although the sample size was relatively large, several limitations should be acknowledged. First, the sample lacked diversity in race, ethnicity, and gender, and was composed entirely of undergraduate students. Findings therefore may not generalize to broader community populations, and future work should attempt to replicate these results in more diverse and clinically relevant samples.

Second, because the study was conducted remotely, we cannot rule out the possibility that participants in the working-memory load condition took a screenshot or photo of the vignette after it was removed from the screen. Although instructions emphasized not doing so, compliance could not be monitored, which may have reduced the intended WM manipulation.

Third, the schizotypal traits measure (SPQ-BR) presents important limitations in this context. The SPQ-BR was not originally validated for use in convenience samples such as undergraduate students, and our sample differed substantially from the population on which the scale was normed. Indeed, the expected three-factor structure (positive, negative, disorganized) did not fit our data well, with fit indices falling below ideal thresholds (CFI = 0.72, RMSEA = 0.09, SMSR = 0.10). Measurement invariance analyses across working-memory load conditions showed only modest changes when moving from configural to metric and from metric to scalar invariance; however, overall model fit remained poor. These issues highlight the need for caution when interpreting trait associations and suggest that future studies should consider alternative schizotypy measures or collect samples with characteristics more aligned with the scale’s validation studies.

Finally, although speech transcripts were manually annotated using a detailed rubric and interrater reliability was acceptable, manual coding introduces the possibility of human bias. Future research employing automated natural language processing (NLP) tools may help reduce subjectivity and further validate inference-generation patterns in both non-clinical schizotypal traits and clinical populations.

### 4.2 Conclusion

To our knowledge, this is the first study to examine inference generation through speech production using manual transcript annotation. Building on these findings, several future directions are warranted. First, validation using a multimethod approach, combining the SPQ-BR with the Multidimensional Schizotypy Scale (or full SPQ) and objective cognitive measures (e.g., WM capacity tasks, beads task), could help triangulate the disorganization–jumping-to-conclusions link. Second, automated natural language processing classifiers, validated against the manual codebook, could scale annotation across samples and extract linguistic markers of disorganization, such as semantic coherence, referential cohesion, and syntactic complexity. Third, integrating computational models of belief updating could quantify the relative weighting of priors versus sensory evidence during narrative generation. Finally, investigating neural correlates, including EEG markers of prediction error or fMRI measures of hierarchical inference, in high versus low disorganization groups during analogous inference tasks could clarify the neurobiological mechanisms underlying these cognitive patterns. Collectively, these approaches would advance our understanding of how disorganized traits influence inference generation and provide a foundation for linking non-clinical schizotypy research to clinical schizophrenia studies.

## Data Availability

The raw data supporting the conclusions of this article will be made available by the authors, without undue reservation.

## Appendix

**Annotation Instructions**

- Create a page break below the clown transcription and copy the 3 page checklist below
- Underline each mentalizing verb as you see it
- Count the total for each word then the overall total
- Go through the checklist and as you read an event described in the transcription highlight the corresponding number on the checklist and then highlight that section in the transcription:

o Highlight visual events in yellow
o Additional visual events highlight yellow and bold text
o Highlight inferred events in green
o Additional inferred events highlight green and bold text

- If the participant describes something that is not listed, add it to the additional events section for its corresponding category
- If they describe something that is clearly not happening, make a note of that
- After annotating for both visual and inferred add up the totals

Example

 alright in this story there's a stranger on the street reading the newspaper and this man comes by and in exchange for the man's newspaper puts his hat on top of him and then he hides his face behind the newspaper and this other man is really confused you know doesn't have really any other immediate reaction except for confusion and this clown walks by and we find out that this clown was hunting down the man with the beard and the hat and [um] to hide himself he replaces his hat with this other man so the clown *thinks* that that stranger is his target so he punches the stranger and then walks away fuming super mad and then the man who stole the strangers newspaper returns the newspaper puts his hat back on and then has to start running away from the other man who is now angry at him for pinning whatever crime he committed on him and making him get punched.

### Figure A. Smoothing Components for Inferred Events

Note: *Tot_inferred* = Inferred events; *possum, negsum, dissum* = positive traits, negative traits and disorganized traits sum scores, respectively.

### Figure B. Smoothing Components for Visual Events

Note: *Tot_visual* = Visual events; *possum, negsum, dissum* = positive traits, negative traits and disorganized traits sum scores, respectively.
